# Sarcoidosis and Cancer: The Role of the Granulomatous Reaction as a Double-Edged Sword

**DOI:** 10.3390/jcm13175232

**Published:** 2024-09-04

**Authors:** Angela Maria Di Francesco, Giuliana Pasciuto, Elena Verrecchia, Ludovico Luca Sicignano, Laura Gerardino, Maria Grazia Massaro, Andrea Urbani, Raffaele Manna

**Affiliations:** 1 Periodic Fever and Rare Diseases Research Centre, Catholic University of Sacred Heart, 00168 Rome, Italy; angelamaria.difrancesco@unicatt.it (A.M.D.F.); mgmassaro91@gmail.com (M.G.M.); 2 Complex Pneumology Operational Unit, A. Gemelli Policlinic Foundation IRCCS, 00168 Rome, Italy; giuliana.pasciuto@policlinicogemelli.it; 3 Department of Aging, Orthopaedic and Rheumatological Sciences, A. Gemelli Policlinic Foundation IRCCS, 00168 Rome, Italy; elena.verrecchia@policlinicogemelli.it (E.V.); ludovicoluca.sicignano@policlinicogemelli.it (L.L.S.); laura.gerardino@policlinicogemelli.it (L.G.); 4 Department of Chemistry, Biochemistry and Molecular Biology, A. Gemelli Policlinic Foundation IRCCS, 00168 Rome, Italy; andrea.urbani@unicatt.it

**Keywords:** sarcoidosis, granulomatous reaction, cancer

## Abstract

**Background/Objectives:** The relationship between sarcoidosis and the occurrence of neoplasia deserves to be investigated, but this relation has been observed in different and heterogeneous populations, leading to conflicting data. To clarify the causal relationship between these two diseases, different risk factors (e.g., smoking), concurrent comorbidities, corticosteroid therapy, and metastasis development—as an expression of cancer aggressiveness—were investigated. **Methods:** In a retrospective study on 287 sarcoidosis outpatients at the Pneumological Department of the Gemelli Foundation (Rome, Italy) between 2000 and 2024, the diagnosis of cancer was recorded in 36 subjects (12.5%). **Results:** The reciprocal timeline of the diseases showed three different scenarios: (1) cancer preceding sarcoidosis or sarcoid-like reactions (63.8%); (2) cancer arising after sarcoidosis diagnosis (8.3%); and (3) sarcoidosis accompanying the onset of malignancy (27.8%). Only two subjects with sarcoidosis and cancer showed metastasis, and one of them was affected by lymphoma. **Conclusions:** These data suggest that granulomatous inflammation due to sarcoidosis may assume an ambivalent role as a “double-edged sword”, according to the M1/M2 macrophage polarization model: it represents a protective shield, preventing the formation of metastasis through the induction of immune surveillance against cancer while, on the other hand, it can be a risk factor for carcinogenesis due to the persistence of a chronic active inflammatory status. Low-dose steroid treatment was administered in only 31.6% of the cancer–sarcoidosis subjects for less than six months to control inflammation activity, with no promotive effect on carcinogenesis observed.

## 1. Introduction

Sarcoidosis is a chronic disease of unknown origin characterized by granulomatous inflammations in the lymph nodes and in virtually all tissues and organs of the body [1]. It can affect people of any age, gender, and racial origin, although its prevalence in women and young people is greater [2].

The etiopathogenesis of sarcoidosis remains elusive, as the exact stimulus that initiates the disease process is not certain; however, there are many hypotheses, with all having a common denominator in the exposure to certain antigens in genetically predisposed individuals. Potential triggers can be PAMPs (pathogen-associated molecular pattern molecules) derived from various infections (*mycobacteria*, *cutibacterium acnes*), antigens of the Kveim–Siltzbach reagent, vimentin, or environmental substances, such as organic dust or inorganic chemicals (Berilllium or Silica) coming from occupational exposure [3,4,5]. Other potential triggers of an exaggerated granulomatous response mimicking sarcoidosis are certain classes of drugs, such as alpha interferon (in hematological patients or those with chronic hepatitis C virus infection) [6], immune checkpoint inhibitors, highly active antiretroviral therapy, and tumor necrosis factor-α antagonists [7,8,9]. These sarcoid reactions that occur in a temporal relationship with the initiation of an offending drug have been named drug-induced sarcoid-like reactions (DISLRs), which tend to spontaneously regress after drug discontinuation. Bone marrow transplantation has been associated with donor-acquired sarcoidosis [10,11]. The association between sarcoidosis and smoking appears complex, with contradictory results reported in different studies, but it appears to be influenced by geographic factors [12,13].

Interestingly, environmental triggers appear to be more important than genetic factors at adult ages. At pediatric ages, prototypical models of sarcoidosis have been described; one of them is the NOD-2-associated Blau syndrome, whereas another that has a sporadic form is early-onset sarcoidosis. Usually, in adults, sarcoidosis appears in the sporadic form but rarely does so in familiar clusters. Indeed, a genetic association has been demonstrated in Löfgren’s syndrome, whereas susceptibility appears to be linked to the major histocompatibility complex (MHC), as suggested by different studies [14,15]. Some identified genes that may be associated with increased susceptibility to sarcoidosis are butyrophilin-like 2 gene (BTNL2), annexin A11 (ANXA11), and angiotensin-converting enzyme (ACE) variants; these associations, however, show high variability across populations [16,17].

From an immunopathogenic point of view, granuloma formation represents a pathological response initiated by CD4 T cells engaged by antigen-presenting cells. The contact with different antigens engages their phagocytosis and presentation by antigen-presenting cells, such as macrophages or dendritic cells, to CD4^+^ T helper lymphocytes. The immune response is then amplified through a highly polarized Th1-type cytokine cascade, such as that of interleukin (IL)-2, tumor necrosis factor (TNF-α), and other players, including T regulatory cells (Tregs), which also produce interferon-gamma (γ-INF) [4,18,19]. The release of γ-IFN and TNF-α, in turn, promotes macrophage accumulation, activation, and aggregation, leading to the development of granulomatous inflammation [20]. Granuloma formation would represent a barrier to the isolation of antigen material. Granulomas show a concentric structure; the internal part is formed by macrophages, epithelioid cells, and giant multinucleated cells. This central area of the granuloma is surrounded by a mixture of CD8- and CD4-positive T lymphocytes, B lymphocytes, monocytes, mast cells, and fibroblasts which, in turn, are surrounded by lamellar rings of hyaline collagen. Overall, the initiation of granuloma formation and the perpetuation of the disease process are characterized by Th1 cytokines, whereas regulatory T cells (Tregs; activating immunoregulation) and the Th17 response have been proposed to play a role in the maintenance of granulomas [21,22].

Sarcoidosis may also be observed in association with neoplasia, but this relation has been investigated in different and heterogeneous populations, leading to conflicting data; moreover, investigative studies have often focused on different types of cancers [23,24,25] without considering the immunosuppressive role of steroid treatment. A retrospective systematic overview of the literature in *Pubmed* over the last thirty years allowed for the extrapolation of at least three causal connections between sarcoidosis and neoplasia (Table 1).

A strong relationship linking chronic active sarcoidosis to malignant lymphoproliferative disease was first highlighted by Brincker [27], who named it sarcoidosis–lymphoma syndrome (the scenario n 1). The activation of the lymphocyte–macrophage axis observed in active sarcoidosis may be the main trigger of the malignant proliferation of lymphoid cells in these subjects [43,44]. This causal evidence has been further reinforced by the recent observation of an increased risk of developing hematological malignancies, especially lymphomas, in sarcoidosis subjects [45]. A second scenario shows an increased risk of associated neoplasia in the form of breast and testicular cancers and lymphoma [26,46,47,48] in sarcoidosis subjects, opening up at least two subsets: (a) the probability of developing cancer in subjects with sarcoidosis could be a consequence of a persistent environmental trigger in a genetically predisposed population; (b) reduced immune surveillance due to immunosuppressive corticosteroid treatment can increase the probability of developing different malignancies. Data on this second clinical scenario are heterogeneous according to the different types of cancer (Hodgkin lymphoma and non-Hodgkin lymphoma are equally represented, in addition to testicular cancer, digestive cancers, breast cancer, and so on) [23,24,25,44,45]. Moreover, while some authors have suggested that sarcoidosis worsens the prognosis of subsequent cancers [49], on the other hand, Chopra and Judson concluded that the available data do not support routine screening for cancer in sarcoidosis subjects [50]. (3) A third scenario of a causal relationship with cancer can occur when sarcoidosis precedes or immediately accompanies the onset of a neoplasm, indicating a particular type of sarcoidosis called sarcoid-like reactions (SLRs) [41,45,51,52]. Moreover, there is recent evidence that oncological immunostimulating therapies induce SLRs, usually within one year from neoplasia. In this scenario, for SLRs, the classification should be drug-induced sarcoidosis when drugs represent the triggering factors of sarcoidosis [53,54].

## 2. Aim

The present work aims to shed light on this complex mosaic through studying the prevalence of cancer in a population at a sarcoidosis-focused day hospital. Moreover, we searched for clinical evidence of subjects who developed antecedent/concurrent/subsequent malignancies. Risk factors (e.g., smoking), concurrent comorbidities, corticosteroid therapy, and metastasis development—as an expression of cancer aggressiveness—were specifically investigated to clarify the relationship between the two diseases.

## 3. Subjects and Methods

A retrospective overview of the literature in *PubMed* from the last thirty years using the keywords “cancer” and “sarcoidosis” was conducted to select the most relevant papers on the subject. In particular, some reviews [45] were interestingly focused on the different possible scenarios that were revealed over the years as more data on this issue and possible causal links started to accumulate.

This investigation was performed on subjects who were routinely referred to a sarcoidosis outpatient/day hospital at the Pneumology Department of the A. Gemelli Foundation of Rome (Italy) from 2000 to 2024.

The definitions used in this report are the following: (1) cancer preceding sarcoidosis was defined when the onset of cancer preceded sarcoidosis by at least 1 year; (2) diagnosis of cancer onset succeeding sarcoidosis; (3) contemporaneity was defined with sarcoidosis simultaneously arising with malignancy (less than 1 year). Within the group of simultaneous diagnoses, subjects had different primary tumors over time: 4 subjects developed more than 1 cancer; only in 1 case did the sarcoidosis develop immediately after the first tumor.

Diagnosis of sarcoidosis was established through a specific diagnostic investigation (BGA, PFT, BAL, CT, or FDG-PET/CT) and a biopsy of the nodules found during the investigation.

All subjects were followed in the same oncological department of the Gemelli Foundation. When necessary, CT or a chest X-ray and PET-CT were performed. ACE and chitotriosidase assays were routinely investigated in the presence of fever and sarcoidosis. Hematological data on CBC were recorded only in the case of abnormal values.

The steroid treatment duration was guided by acute-phase reactants and symptoms, and it was always brief and less than 6 months. Follow-up was conducted every six to twelve months through clinical and functional assessments.

The clinical data were retrospectively collected from the electronic informatic system of the Gemelli Foundation with respect for privacy since 2000. The follow-up of all cancer patients was updated until July 2024. The following markers or parameters were recorded: type and date of chemotherapy, presence of metastases and their localization, smoking habits, presence of fever during sarcoidosis diagnosis, comorbidities, and presence and dose of corticosteroid treatment. The onset of cancer and sarcoidosis and the number of sarcoidosis subjects with or without cancer were accurately recorded.

Statistical analysis was performed with a *t*-test, with a *p*-value of <0.05 indicating statistically significant results.

## 4. Results

A total of 287 sarcoidosis subjects were identified (161 females and 126 males, F:M = 1.3, mean age: 61.5 years), of whom 36 were associated with a cancer diagnosis (28 females and 8 males, F:M = 3.5). The mean age of the first tumor diagnosis was 54.4 ± 12.0 years; the mean age of sarcoidosis diagnosis was 56.4 ± 12.0 years. The average follow-up duration was 78.1 ± 62.7 months. The population of 36 sarcoidosis–cancer subjects was heterogeneous, with 11 breast cancer subjects (30.5%), four melanoma subjects (11.1%), four Hodgkin subjects and one Waldenstrom macroglobulinemia subject (a total of five subjects, 13.9%), three thyroid cancer subjects (8.3%), three endometrial cancer subjects (8.3%), two ovarian cancer subjects (5.5%), and other types of tumors (Table 2).

The prevalence of gynecological cancers (47.2% of the total sarcoidosis population) was due to the sex distribution in the studied population (77.8% female with respect to male).

Steroid treatment was administered only in one-third of the cases in our study group, with the same percentage in all subsets (Table 3).

Different comorbidities are reported in Table 4.

**Figure 1:**
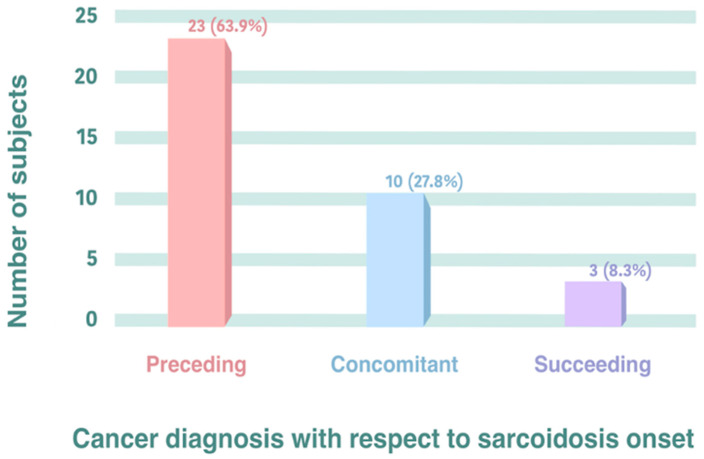
Cancer subjects (36 out of 287) distributed with respect to temporal relationship between cancer and sarcoidosis onset.

Only two subjects with sarcoidosis and cancer showed metastasis, and one of them was affected by lymphoma. Regarding the timing of cancer and sarcoidosis onset, 63.9% of the subjects in the present study showed cancer preceding the sarcoidosis diagnosis, while 27.8% showed sarcoidosis and cancer during the same period (concomitant), and only 8.3% showed cancer after the sarcoidosis diagnosis (Figure 1).

**Figure 2:**
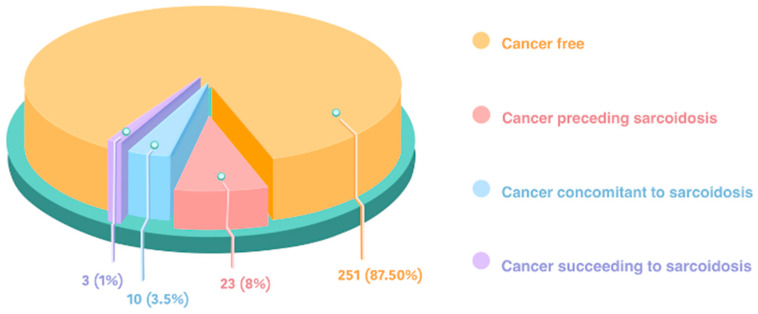
Percentage of subjects with and without cancer in the total sarcoidosis population (287 subjects). The temporal relationship between cancer and sarcoidosis onset is indicated in the cancer populations.

Cancer was diagnosed in 36 subjects (12.5%) of the total sarcoidosis population (287 subjects); it preceded in 8% and was concomitant in 3.5% of the subjects, but it followed sarcoidosis only in 1% of the total sarcoidosis population (a rare event) (Figure 2). Four subjects with sarcoidosis showed multiple cancers.

One subject developed an IgM gammopathy after sarcoidosis diagnosis, which slowly evolved over 10 years towards an overt Waldenstrom macroglobulinemia.

The *t*-test on the age of the Hodgkin lymphoma subjects (mean age: 42.2 ± 8.0) vs. the cancer–sarcoidosis population (mean age 55.9 ± 17.0) demonstrated a significant correlation: *p* < 0.015.

In the group of sarcoidosis and neoplasia subjects, one subject had skin localization (who developed Waldenstrom), two had erythema nodosum, and another had arthralgias in undifferentiated connective tissue disease with MGUS. Seven subjects had extrapulmonary (abdominal) lymph node localizations. The other 22 subjects did not have extrapulmonary and extra-lymph node localizations, such as uveitis and arthritis.

Among the subjects with sarcoidosis and cancer, only four had fever, and smoking habits were present in 85 sarcoidosis subjects and in 13 subjects in the sarcoidosis + cancer group; the *p*-value was not significant (see Supplementary Table S1). The data on ACE and chitotriosidase assays and CBC are not included in the results because statistical significance between different groups was not achieved. We observed the appearance of sarcoidosis in 15/36 subjects after different chemotherapies (CHTs); 3/15 were treated with antiestrogen (AE) drugs, 10/15 were treated with multiple CHTs, 2/15 were treated with CHT + AE, and one of them developed sarcoidosis after pembrolizumab.

## 5. Discussion

When investigating sarcoidosis, there is often a risk of selection bias; indeed, many studies addressing the cancer–sarcoidosis connection have been conducted on hospitalized sarcoidosis subjects, leading to the probability of bias due to the presence of diseases other than cancer [50]. Then, the question of whether sarcoidosis might play a protective role, rather than representing a risk factor for the development of neoplasia, is still considered to be open [55,56].

The strength of the present series is that it comes from a specific outpatient clinic for sarcoidosis subjects and does not have the bias observed among inpatients. We observed three different subsets: (1) the diagnosis of cancer preceded that of sarcoidosis in 63.9% of the cases; (2) cancer and sarcoidosis diagnoses were simultaneous in 27.8% of the cases; (3) cancer succeeded sarcoidosis in 8.3% of the cases. In one case, sarcoidosis was diagnosed after the use of pembrolizumab for breast cancer while, in another, it was diagnosed after methotrexate therapy for choriocarcinoma (cancer preceding sarcoidosis); in a third case, it was diagnosed after chemotherapy (ABVD) plus COVID-19 vaccination. In our series, we observed a subset of 13.9% (5/36) with lymphoma (four Hodgkin and one Waldenstrom). All Hodgkin subjects preceded sarcoidosis by 3 years, and three of them reached remission, while one had recurrence after 17 months. The Hodgkin lymphoma subjects (mean age: 42.2 ± 8.0) were significantly younger (*p* < 0.015) than the other cancer subjects (mean age: 55.9 ± 17.0), according to a *t*-test.

The observation that the immune system is involved in both cancer and sarcoidosis addresses the question of possible links between them. Indeed, the following data have been reported on immune dysregulation and chronic inflammation in vitro: (a) myeloid dendritic cell dysfunction possibly leading to decreased tumor immune surveillance [12,57]; (b) an increase in mitotic activity and uncontrolled cellular proliferation [58,59]; and (c) uncontrolled production of inflammatory cytokines (e.g., tumor necrosis factor-α, interleukin-6 and transforming growth factor-β, nitric oxide, and vascular endothelial growth factor) which, in turn, may promote angiogenesis, cellular proliferation, stromal growth, and tissue remodeling and may further increase the risk of malignancy [59,60,61]. Macrophage polarization is the common denominator of inflammation and tumor progression, since it is considered a key player in immune processes, engaging both the innate immune response and adaptive immunity [62]. The polarization status in the macrophage M1-like (“killer” macrophages) and M2-like (“healer” macrophage) axis in humans is considered a dichotomy underlying the balance between homeostasis and chronic inflammation and disease (disequilibrium) [63]. However, the M1/M2 paradigm applies to a “continuum” of activation states playing an important dynamic role during inflammation and its resolution. A fine-tuned balance and switching back and forth between the M1 and M2 polarization states are necessary to allow the beneficial processes of stress, inflammation, resolution, and repair.

**Figure 3:**
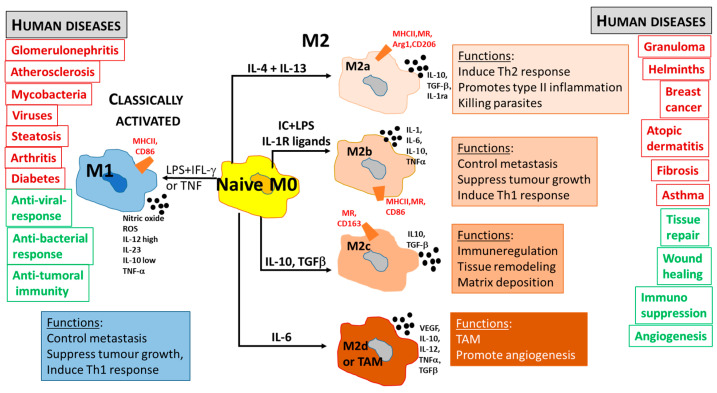
Summary of the main macrophage polarization states of activated macrophages in relation to functional roles and human diseases. M1-like or M2-like activation states can be induced by different stimuli and signaling pathways. In humans, distinct defensive or healing schemas are reported to be related to M1-like or M2-like polarization. Inducing factors are indicated on the arrows; antigens and receptors expression is represented in red; cytokines and chemokines production is shown in bold. LPS: lipopolysaccharide; MR: mannose receptor; TNF: tumor necrosis factor; TLR: Toll Like Receptor; Arg 1: arginase 1; IFNγ: interferon gamma; IL: interleukin; MCP: monocyte chemoattractant protein; TGF: transforming growth factor; MCSF: macrophage colony stimulating-factor; ROS: reactive oxygen species; MHC: major histocompatibility complex; VEGF: vascular endothelial growth factor. Modified from J. Novais Barbosa, D. Pereira Vasconcelos, Macrophage response to biomaterials, Handbook of Biomaterials Biocompatibility, 2020 [68].

The inflammatory environment is dominated by a wide range of different Toll-like receptors (TLR 1–9) and IFN signaling. The first line of defense against intracellular pathogens is represented by classically activated or M1 macrophages, which exhibit a high level of phagocytic activity and promote the Th1 polarization of CD4 cells. Depending on the nature of the M1 stimulus, a different level of expression of CD64 and CD80 markers has been described. They produce proinflammatory mediators, such as cytokines, chemokines, and reactive oxygen and nitrogen intermediates, which induce the activation of various antimicrobial mechanisms. The Th1 response activation and complement-mediated phagocytosis lead to pathogen killing, the final resolution of inflammation [64], and cancer cell cytolysis. However, in order to prevent tissue damage to the host and avoid severe immunopathologies, such responses must be controlled by M2-polarized macrophages (CD163^+^) through the production of anti-inflammatory cytokine mediators [65,66], as is common in sarcoidosis. There are subpopulations of polarized M2-like (M2a, M2b, M2c, and M2d or TAM) macrophages that are particularly involved during parasitic, helminthic, and fungal infections. Unlike classically activated M1-like macrophages, M2-like macrophages play a modulatory role, inducing the production of anti-inflammatory mediators such as IL-4, IL-10, and TGF-β [67]. Further, M2-like macrophages are highly endocytic and partially phagocytic and are involved in a variety of functions, such as repair mechanisms, metabolic processes, and different pathogenetic pathways that evolve toward granuloma formation (Figure 3).

It is important to point out that a strictly clear-cut dichotomy is not always observed during infections; rather, each pathogen promotes a “tailored” inflammation. The spatiotemporal orchestration of the resolution of the inflammation process can take minutes to a few days to resolve minor damage (acute inflammation); otherwise, major damage can engage excessive or subnormal inflammatory responses, which can be prolonged for months to years, causing non-resolving inflammation, such as in cancer, inflammatory autoimmune diseases, or chronic inflammation due to infection. In cancer, inflammation is important for tumor progression, and the predominance of either M1-like or M2-like populations has been described in different tumors [69,70]. The development of a sufficient and adequate type 1 immune response, where macrophages and lymphocytes may play a regulatory and protective role, is pivotal for eliciting anti-inflammatory mechanisms that are necessary to suppress inflammation in tissue, promote remodeling, retain homeostasis, and assure the survival of the host [71]. Heterogeneous TAMs, through the regulation of their own polarization profile triggered by different exogenous or endogenous stimuli and through reprogramming and continuous plasticity, can either be the initiators of the inflammatory response or participate in its resolution and the maintenance of homeostasis. TAMs arise from adult myeloid precursors found in circulation; in cases involving bone marrow transplantation, macrophages associated with lymphoma also develop from these bone marrow precursors. Most TAMs are thought to resemble M2 macrophages; they express higher levels of anti-inflammatory cytokines and angiogenic factors than their M1-polarized counterparts. They can reprogram the immunosuppressive microenvironment and promote the proliferation, invasion, and metastasis of cancer cells [72]. Furthermore, macrophages and M2 polarization may induce the transition to fibrosis in the advanced disease stage of sarcoidosis [73,74]. The causal relationships between sarcoidosis and cancer can, therefore, be reviewed on the basis of the common denominator of the inflammation status and upon the dynamic and continual M1–M2 switch. Indeed, the administration of ibiquimod (a TLR 7/8 agonist) induces an M1 re-education and enhances the development of the anticancer microenvironment [75].

In our series, cancer arose after the diagnosis of sarcoidosis in only 8.3% of cases; the low rate of this subset is consistent with the observation that a significant association between sarcoidosis and malignancy was excluded by a systematic review after accounting for possible detection biases and publication biases [31]. A meta-analysis of 16 cohorts and case–control studies reported a moderate association between sarcoidosis and malignancy [32]; however, this meta-analysis had severe limitations related to heterogeneity in sample sizes and designs, as well as the case selection criteria; further, the data were retrieved through record linkages between various healthcare databases, such as hospital discharge datasets and national registers, which usually do not report information on relevant covariates or clinical details. As a consequence, information on organ-specific involvements in sarcoidosis, as well as relevant confounding or modifying factors, such as smoking habits and previous immunosuppressive therapies, could not be evaluated. A concomitant review also argued that, apart from the risk of hematological diseases, it is not possible to establish the existence of a net risk for neoplasia in sarcoidosis; only the association with scleroderma appears to be clearly defined [50].

However, in our series, strictly based on homogenous criteria, the most prevalent subset (23/36) was that in which cancer preceded sarcoidosis (63.9%); sarcoidosis developed after the diagnosis of a tumor or in conjunction with it, emphasizing the difference between “true” sarcoidosis and SLRs, a secondary event triggered by neoplastic proliferation [41,76,77] or in response to chemotherapeutic agents that increased immunoreactivity, such as interferon or PDL1 inhibitors [78,79,80] to counteract neoplastic proliferation. During a long follow-up period (78.1 ± 62.7 months), only two subjects with cancer had a recurrence of the disease (one of them was affected by lymphoma), which is consistent with the observation of low incidence of cancer after sarcoidosis [31,81]. SLRs may be considered a marker of a strong immune response and may play a barrier role against cancer cells [82,83,84] and improve the prognosis. Two of the 36 subjects belonged to the same family (father and daughter) in a family cluster; the father developed Waldenstrom’s macroglobulinemia many years after the onset of sarcoidosis, while the daughter developed sarcoidosis two years after an ovarian cancer diagnosis. Furthermore, in one patient, sarcoidosis developed several months before cancer relapse, playing a sentinel role during the follow-up. Overall, all four subjects with sarcoidosis who showed multiple cancers over time did not develop metastasis.

Eventually, in these series, the steroid treatment was always found to be brief and less than 6 months; it was administered in only eight cases (31.6%), a restricted group, with a similar percentage to that in the preceding subset, indicating that this treatment was only aimed at controlling inflammatory activity; therefore, it did not have an immunosuppressive or promotive effect on carcinogenesis.

At present, the differentiation between “true” sarcoidosis and SLRs triggered by malignancy relies only upon clinical findings; that is, SLRs lack other common and specific clinical features of typical sarcoidosis. This hypothesis is consistent with the prevalence of sarcoidosis among juveniles and females. Indeed, our data confirmed that there was a female/male ratio of 1.3 in the global sarcoidosis population and 3.5 in the subgroup of cancer–SLR subjects. Brito-Zerón et al. [85] have observed that the frequency of immune-mediated diseases (IMDs) in a group of subjects with sarcoidosis was 1.64-fold higher than that reported in the general population. They concluded that women with sarcoidosis have a two-fold higher frequency of concomitant IMDs, as sarcoidosis was observed to be more prevalent in women than in men in some studies [86].

## 6. Take-Home Messages

Clinicians should be aware that sarcoidosis and cancer can coexist and that granulomatous reactions are not uncommon in the course of solid neoplasia and hematological malignancies. Furthermore, cancer treatments, such as immunotherapies, may induce SLRs, as already observed with some therapeutic approaches (e.g., interferon, Pd-1, and PdL-1 inhibitors) [78]. However, differentiating sarcoidosis from cancer-associated granulomatosis is not easy, due to their similar histological features; moreover, ^18^FDG PET-CT is used more and more frequently, whereas the biopsy [87,88] remains the gold standard for differentiating sarcoidosis lymph nodes from metastatic ones.

The results of this investigation support the conclusion that sarcoidosis is almost a benign disease, but it plays an ambivalent role as a “double-edged sword”: on one hand, its protective role is characterized by the fact that sarcoidosis can limit cancer progression and may be considered as a model of a natural immune barrier to tumors; on the other hand, the prolonged immune overdrive with M2 macrophages can lead to hematological lymphoproliferation, such as lymphoma, MGUS, and macroglobulinemia, according to Brincker’s observation [26,27]. Then, M1 and M2 macrophage polarization represents an inflammatory microenvironment [89], in which M1 macrophages have anticancer properties in SLRs [90], while M2 macrophages can induce lymphoproliferation. Future drugs promoting M1 re-education, as data on ibiquimod already suggest [75], will result in new anticancer strategies.

Finally, prolonged corticosteroid treatment has an immunodepressive effect and is, thus, a risk factor for tumor development. Therefore, it should be used for a minimal duration to control sarcoidosis symptoms.

## Figures and Tables

**Table 1:** Causal links between cancer and sarcoidosis.

Timing	Evidence-Based Associations	Reported Data	Rationale	References
Sarcoidosis (several years before) ↓ Lymphoma	Sarcoidosis-lymphoma syndrome.	An increased risk of cancer (by 30–40%) was observed in skin cancers, hematological malignancies, and leukemias.	Elevation of pro-proliferative cytokines such as BAFF for B lymphocytes could be a possible explanation for the emergence of clonal proliferation in sarcoidosis subjects in comparison with other autoimmune diseases.	[12,26,27,28]
Sarcoidosis ↓ Cancer	Increased risk of neoplasia in sarcoidosis subjects—paraneoplastic sarcoidosis.	Contradictory data according to the different types of cancer (lymphoma, testicular cancer, digestive cancers, breast cancer, and so on).	(A) A persistent environmental trigger in a genetically predisposed patient can increase the probability of developing both sarcoidosis and cancer. (B) Steroid-related immunosuppression can increase the risk of neoplasia.	[29,30,31,32,33,34]
Cancer ↓ Sarcoidosis- Like Reactions	Sarcoidosis-like reactions (SRLs)	(A) The presence of cancer-associated reactions (SLRs) could be a marker of good prognosis, indicating a strong immune response to tumor cells. (B) Induced by sarcoidosis drugs (TCZ, PDL1, TNFi, and so on). (C) Sarcoidosis in allogenic (D) or autologous bone marrow transplantation: “donor-acquired sarcoidosis”.	The modulation of the immune system due to immunotherapies, the presence of cancer, or as a consequence of bone marrow transplantation may explain SLRs that are mainly diagnosed within 1 year from the trigger.	[10,11,26,27,35,36,37,38,39,40,41,42]

**Table 2:** Subjects divided by cancer type.

Cancer	Number of Subjects	Sex ^1^ F:M	Age ^2^ (Year)
Breast	11	11:0	50.5 ± 13.0
Melanoma	4	2:2	54.5 ± 5.0
Lymphoma di Hodgkin ^3^	4	3:1	44.0 ± 5.0
Waldenstrom’s macroglobulinemia	1	0:1	75
Thyroid	3	3:0	55.0 ± 14.0
Endometrial cancer	3	3:0	57.7 ± 6.0
Ovary cancer	2	2:0	56.0 ± ^4^ 6.0
Kidney	1	0:1	66
Adenocarcinoma	1	1:0	68
Choriocarcinoma	1	1:0	56
Cervical cancer	1	1:0	53
Rectum	1	0:1	72
Myeloproliferative neoplasm	1	1:0	78
Seminoma	1	0:1	42
Urothelial	1	0:1	58

^1^ ratio of female over male is indicated; ^2^ age is in years ± SD; ^3^ *t*-test for Lymphoma di Hodgkin vs. all other cancer *p* = 0.015; ^4^ SE.

**Table 3:** Subjects were grouped according to the timeline of cancer and sarcoidosis diagnoses.

Cancer-Sarcoidosis ^1^	N Subjects	ΔYrs ^2^	%	Mean Age at Cancer Onset (Years)	Mean Age at Sarcoidosis Onset (Years)	F:M	Steroid Treatment
Preceding	23	2.1	63.9	54.7 ± 11.0	56.8 ± 11.0	17:6	8 *
Concomitant	10	0.1	27.8	57.4 ± 13.0	57.5 ± 13.0	10:0	3
Posterior	3	6	8.3	67.7 ± 7.0	61.7 ± 3.0	1:2	1

^1^ anterior, concomitant and posterior was considered cancer diagnosis timing with respect to sarcoidosis onset. ^2^ Difference in years between the mean ages of the two diagnosis onsets. * One out of eight subjects took steroid therapy for 6 consecutive months before sarcoidosis onset.

**Table 4:** Comorbidities observed.

Comorbidity ^1^	Number of Subjects
Hypertension	8
Diabetes	4
Liver steatosis	2
Dyslipidemia	2
Autoimmunity (thyroid ^2^, lichen and mixed connectivitis)	6
Allergy ^3^	2
Cutaneous Mycosis, Acne	2
Favism	1
Osteoporosis	2
Arthrosis	2
Guillain-Barré Syndrome	1
Prostatic hypertrophy	1
Migraine	1
Peripheral venous insufficiency	1
Ischemic Heart disease	1
Sciatica	1
Depression	1
None	7

^1^ More of one pathology was shown by the majority of our subjects. ^2^ Thyroid pathologies: hypothyroidism, thyroiditis, thyroid adenoma. ^3^ Allergy: pollen, nickel, ibuprofen.

## Data Availability

All data are shown in this paper.

## Supplementary Materials

The following supporting information can be downloaded at: https://www.mdpi.com/article/10.3390/jcm13175232/s1, Table S1: Smoking habits and fever.

## List of Abbreviations

BAL	Broncho Alveolar Lavage
BGA	Blood Gas Analysis
CBC	Cell Blood Count
CT	Computerized Tomography
IMDs	immune-mediated diseases
PFT	Pulmonary Function Test
FDG-PET/CT	^18^Fluorodessossiglucose Positron Emission Tomography/Computerized Tomography
SLR	sarcoid-like reaction
TAMs	Tumor Associated Macrophages

## References

[1] Iannuzzi M.C., Rybicki B.A., Teirstein A.S. (2007). Sarcoidosis. N. Engl. J. Med..

[2] Lazarus A. (2009). Sarcoidosis: Epidemiology, etiology, pathogenesis, and genetics. Dis. Mon..

[3] Culver D.A. (2016). Beryllium disease and sarcoidosis: Still besties after all these years?. Eur. Respir. J..

[4] Sakthivel P., Bruder D. (2017). Mechanism of granuloma formation in sarcoidosis. Curr. Opin. Hematol..

[5] Eberhardt C., Thillai M., Parker R., Siddiqui N., Potiphar L., Goldin R., Timms J.F., Wells A.U., Kon O.M., Wickremasinghe M. (2017). Proteomic Analysis of Kveim Reagent Identifies Targets of Cellular Immunity in Sarcoidosis. PLoS ONE.

[6] Ramos-Casals M., Mañá J., Nardi N., Brito-Zerón P., Xaubet A., Sánchez-Tapias J.M., Cervera R., Font J. (2005). HISPAMEC Study Group. Sarcoidosis in patients with chronic hepatitis C virus infection: Analysis of 68 cases. Medicine.

[7] Ramos-Casals M., Perez-Alvarez R., Perez-de-Lis M., Xaubet A., Bosch X. (2011). BIOGEAS Study Group. Pulmonary disorders induced by monoclonal antibodies in patients with rheumatologic autoimmune diseases. Am. J. Med..

[8] Chanson N., Ramos-Casals M., Pundole X., Suijkerbuijk K., José de Barros E., Silva M., Lidar M., Benesova K., Leipe J., Acar-Denizli N. (2021). Immune checkpoint inhibitor-associated sarcoidosis: A usually benign disease that does not require immunotherapy discontinuation. Eur. J. Cancer.

[9] Kim S.T., Pundole X., Dadu R., Lambotte O., Ramos-Casals M., Suarez-Almazor M.E. (2021). Use of immune checkpoint inhibitors in cancer patients with pre-existing sarcoidosis. Immunotherapy.

[10] Heyll A., Meckenstock G., Aul C., Söhngen D., Borchard F., Hadding U., Mödder U., Leschke M., Schneider W. (1994). Possible transmission of sarcoidosis via allogeneic bone marrow transplantation. Bone Marrow Transplant..

[11] Schattenberg A.V., Baynes C., van Dijk M.C., Koster A., van Cleef P.H., Preijers F.W., Hermus A., Raemaekers J.M. (2006). A mediastinal mass after donor lymphocyte infusion for relapse of chronic myeloid leukemia after allogeneic stem cell transplantation. Leuk. Lymphoma.

[12] Landgren O., Engels E.A., Pfeiffer R.M., Gridley G., Mellemkjaer L., Olsen J.H., Kerstann K.F., Wheeler W., Hemminki K., Linet M.S. (2006). Autoimmunity and susceptibility to Hodgkin lymphoma: A population-based case–control study in Scandinavia. J. Natl. Cancer Inst..

[13] Le Jeune I., Gribbin J., West J., Smith C., Cullinan P., Hubbard R. (2007). The incidence of cancer in patients with idiopathic pulmonary fibrosis and sarcoidosis in the UK. Respir. Med..

[14] Ji J., Shu X., Li X., Sundquist K., Sundquist J., Hemminki K. (2009). Cancer risk in hospitalized sarcoidosis patients: A follow-up study in Sweden. Ann. Oncol..

[15] Kristinsson S., Landgren O., Sjöberg J., Turesson I., Björkholm M., Goldin L. (2009). Autoimmunity and risk for Hodgkin’s lymphoma by subtype. Haematologica.

[16] Fischer A., Rybicki B.A. (2015). Granuloma genes in sarcoidosis: What is new?. Curr. Opin. Pulm. Med..

[17] Crouser E.D., Culver D.A., Knox K.S., Julian M.W., Shao G., Abraham S., Liyanarachchi S., Macre J.E., Wewers M.D., Gavrilin M.A. (2009). Gene expression profiling identifies MMP-12 and ADAMDEC1 as potential pathogenic mediators of pulmonary sarcoidosis. Am. J. Respir. Crit. Care Med..

[18] Broos C.E., Hendriks R.W., Kool M. (2016). T-cell immunology in sarcoidosis: Disruption of a delicate balance between helper and regulatory T-cells. Curr. Opin. Pulm. Med..

[19] Ramstein J., Broos C.E., Simpson L.J., Ansel K.M., Sun S.A., Ho M.E., Woodruff P.G., Bhakta N.R., Christian L., Nguyen C.P. (2016). IFN-γ-Producing T-Helper 17.1 Cells Are Increased in Sarcoidosis and Are More Prevalent than T-Helper Type 1 Cells. Am. J. Respir. Crit. Care Med..

[20] Chen E.S., Moller D.R. (2011). Sarcoidosis—Scientific progress and clinical challenges. Nat. Rev. Rheumatol..

[21] Mellemkjaer L., Pfeiffer R.M., Engels E.A., Gridley G., Wheeler W., Hemminki K., Olsen J.H., Dreyer L., Linet M.S., Goldin L.R. (2008). Autoimmune disease in individuals and close family members and susceptibility to non-Hodgkin’s lymphoma. Arthritis Rheum..

[22] Shevach E.M. (2000). Regulatory T cells in autoimmmunity. Annu. Rev. Immunol..

[23] Dick J., Begent R.H., Meyer T. (2010). Sarcoidosis and testicular cancer: A case series and literature review. Urol. Oncol..

[24] Wenter V., Albert N.L., Ahmaddy F., Unterrainer M., Hornung J., Ilhan H., Bartenstein P., Spitzweg C., Kneidinger N., Todica A. (2021). The diagnostic challenge of coexistent sarcoidosis and thyroid cancer—A retrospective study. BMC Cancer.

[25] Grados A., Ebbo M., Bernit E., Veit V., Mazodier K., Jean R., Coso D., Aurran-Schleinitz T., Broussais F., Bouabdallah R. (2015). Sarcoidosis Occurring After Solid Cancer: A Nonfortuitous Association: Report of 12 Cases and Review of the Literature. Medicine.

[26] Brincker H. (1972). Sarcoid reactions and sarcoidosis in Hodgkin’s disease and other malignant lymphomata. Br. J. Cancer.

[27] Brincker H. (1986). The sarcoidosis-lymphoma syndrome. Br. J. Cancer.

[28] Brincker H. (1989). Coexistence of sarcoidosis and malignant disease: Causality or coincidence?. Sarcoidosis.

[29] Askling J., Grunewald J., Eklund A., Hillerdal G., Ekbom A. (1999). Increased risk for cancer following sarcoidosis. Am. J. Respir. Crit. Care Med..

[30] Boffetta P., Rabkin C.S., Gridley G. (2009). A cohort study of cancer among sarcoidosis patients. Int. J. Cancer.

[31] Ungprasert P., Crowson C.S., Matteson E.L. (2017). Risk of Malignancy Among Patients with Sarcoidosis: A Population-Based Cohort Study. Arthritis Care Res..

[32] Bonifazi M., Bravi F., Gasparini S., La Vecchia C., Gabrielli A., Wells A.U., Renzoni E.A. (2015). Sarcoidosis and cancer risk: Systematic review and meta-analysis of observational studies. Chest.

[33] Spiekermann C., Kuhlencord M., Huss S., Rudack C., Weiss D. (2017). Coexistence of sarcoidosis and metastatic lesions: A diagnostic and therapeutic dilemma. Oncol. Lett..

[34] Søgaard K.K., Sværke C., Thomsen R.W., Nørgaard M. (2015). Sarcoidosis and subsequent cancer risk: A Danish nationwide cohort study. Eur. Respir. J..

[35] Rubio-Rivas M., Moreira C., Marcoval J. (2020). Sarcoidosis related to checkpoint and BRAF/MEK inhibitors in melanoma. Autoimmun. Rev..

[36] Steinfort D.P., Irving L.B. (2009). Sarcoidal reactions in regional lymph nodes of patients with non-small cell lung cancer: Incidence and implications for minimally invasive staging with endobronchial ultrasound. Lung Cancer.

[37] Padilla M.L., Schilero G.J., Teirstein A.S. (2002). Donor-acquired sarcoidosis. Sarcoidosis Vasc. Diffus. Lung Dis..

[38] Morita R., Hashino S., Kubota K., Onozawa M., Kahata K., Kondo T., Suzuki S., Matsuno Y., Imamura M., Asaka M. (2009). Donor cell-derived sarcoidosis after allogeneic BMT. Bone Marrow Transplant..

[39] Kushima H., Ishii H., Ikewaki J., Takano K., Ogata M., Kadota J. (2013). Sarcoidosis in donor-derived tissues after haematopoietic stem cell transplantation. Eur. Respir. J..

[40] Puzanov I., Diab A., Abdallah K., Bingham C.O., Brogdon C., Dadu R., Hamad L., Kim S., Lacouture M.E., LeBoeuf N.R. (2017). Society for Immunotherapy of Cancer Toxicity Management Working Group. Managing toxicities associated with immune checkpoint inhibitors: Consensus recommendations from the Society for Immunotherapy of Cancer (SITC) Toxicity Management Working Group. J. Immunother. Cancer.

[41] Tchernev G., Tana C., Schiavone C., Cardoso J.C., Ananiev J., Wollina U. (2014). Sarcoidosis vs. Sarcoid-like reactions: The Two Sides of the same Coin?. Wien Med Wochenschr.

[42] Gebrekidan S., Schaller T., Rank A., Kircher M., Lapa C. (2021). Sarcoid-like reactions: A potential pitfall in oncologic imaging. Eur. J. Nucl. Med. Mol. Imaging.

[43] Chalayer É., Bachy E., Occelli P., Weiler L., Faurie P., Ghesquieres H., Pavic M., Broussolle C., Sève P. (2015). Sarcoidosis and lymphoma: A comparative study. QJM.

[44] London J., Grados A., Fermé C., Charmillon A., Maurier F., Deau B., Crickx E., Brice P., Chapelon-Abric C., Haioun C. (2014). Sarcoidosis occurring after lymphoma: Report of 14 patients and review of the literature. Medicine.

[45] El Jammal T., Pavic M., Gerfaud-Valentin M., Jamilloux Y., Sève P. (2020). Sarcoidosis and Cancer: A Complex Relationship. Front. Med..

[46] de Charry F., Sadoune K., Sebban C., Rey P., de Parisot A., Nicolas-Virelizier E., Belhabri A., Ghesquières H., Ninet J., Faurie P. (2016). Association lymphome et granulomatose: À propos d’une série de cas [Association of lymphoma and granulomatosis: A case series]. Rev. Med. Interne.

[47] Arish N., Kuint R., Sapir E., Levy L., Abutbul A., Fridlender Z., Laxer U., Berkman N. (2017). Characteristics of Sarcoidosis in Patients with Previous Malignancy: Causality or Coincidence?. Respiration.

[48] Kaikani W., Boyle H., Chatte G., de la Roche E., Errihani H., Droz J.P., Fléchon A. (2011). Sarcoid-like granulomatosis and testicular germ cell tumor: The ‘Great Imitator’. Oncology.

[49] Shu X., Ji J., Sundquist K., Sundquist J., Hemminki K. (2011). Survival in cancer patients with previous hospitalization for sarcoidosis: A Swedish population-based cohort study during 1964-2006. Ann. Oncol..

[50] Chopra A., Judson M.A. (2015). How are cancer and connective tissue diseases related to sarcoidosis?. Curr. Opin. Pulm. Med..

[51] Apalla Z., Kemanetzi C., Papageorgiou C., Bobos M., Manoli M., Fotiadou C., Hatzibougias D., Boukovinas I., Stergiou E., Levva S. (2021). Challenges in sarcoidosis and sarcoid-like reactions associated to immune checkpoint inhibitors: A narrative review apropos of a case. Dermatol. Ther..

[52] Park S.D., Kim M.S., Han M.H., Kim Y.J., Jung H.Y., Choi J.Y., Cho J.H., Park S.H., Kim C.D., Kim Y.L. (2023). Renal Sarcoidosis-like Reaction Induced by PD-1 Inhibitor Treatment in Non-Small Cell Lung Cancer: A Case Report and Literature Review. Medicina.

[53] Chopra A., Nautiyal A., Kalkanis A., Judson M.A. (2018). Drug-Induced Sarcoidosis-Like Reactions. Chest.

[54] Miedema J., Nunes H. (2021). Drug-induced sarcoidosis-like reactions. Curr. Opin. Pulm. Med..

[55] Bonifazi M., Renzoni E.A., Lower E.E. (2021). Sarcoidosis and maligancy: The chicken and the egg?. Curr. Opin. Pulm. Med..

[56] Hachisu Y., Koga Y., Kasama S., Kaira K., Uno S., Yatomi M., Aoki-Saito H., Tsurumaki H., Jingu A., Sunaga N. (2022). The Relationship between Tumor Development and Sarcoidosis in Aspects of Carcinogenesis before and after the Onset of Sarcoidosis. Medicina.

[57] Basbous S., Levescot A., Piccirilli N., Brizard F., Guilhot F., Roy L., Bourmeyster N., Gombert J.M., Herbelin A. (2016). The Rho-ROCK pathway as a new pathological mechanism of innate immune subversion in chronic myeloid leukaemia. J. Pathol..

[58] Zaba L.C., Smith G.P., Sanchez M., Prystowsky S.D. (2010). Dendritic cells in the pathogenesis of sarcoidosis. Am. J. Respir. Cell Mol. Biol..

[59] Tana C., Giamberardino M.A., Di Gioacchino M., Mezzetti A., Schiavone C. (2013). Immunopathogenesis of sarcoidosis and risk of malignancy: A lost truth?. Int. J. Immunopathol. Pharmacol..

[60] Sekiya M., Ohwada A., Miura K., Takahashi S., Fukuchi Y. (2003). Serum vascular endothelial growth factor as a possible prognostic indicator in sarcoidosis. Lung.

[61] Pabst S., Karpushova A., Diaz-Lacava A., Herms S., Walier M., Zimmer S., Cichon S., Nickenig G., Nöthen M.M., Wienker T.F. (2010). VEGF gene haplotypes are associated with sarcoidosis. Chest.

[62] Atri C., Guerfali F.Z., Laouini D. (2018). Role of Human Macrophage Polarization in Inflammation during Infectious Diseases. Int. J. Mol. Sci..

[63] Tarique A.A., Logan J., Thomas E., Holt P.G., Sly P.D., Fantino E. (2015). Phenotypic, functional, and plasticity features of classical and alternatively activated human macrophages. Am. J. Respir. Cell Mol. Biol..

[64] Mantovani A., Sica A., Sozzani S., Allavena P., Vecchi A., Locati M. (2004). The chemokine system in diverse forms of macrophage activation and polarization. Trends. Immunol..

[65] Italiani P., Mazza E.M., Lucchesi D., Cifola I., Gemelli C., Grande A., Battaglia C., Bicciato S., Boraschi D. (2014). Transcriptomic profiling of the development of the inflammatory response in human monocytes in vitro. PLoS ONE.

[66] Arnold L., Henry A., Poron F., Baba-Amer Y., van Rooijen N., Plonquet A., Gherardi R.K., Chazaud B. (2007). Inflammatory monocytes recruited after skeletal muscle injury switch into antiinflammatory macrophages to support myogenesis. J. Exp. Med..

[67] Ambarus C.A., Noordenbos T., de Hair M.J., Tak P.P., Baeten D.L. (2012). Intimal lining layer macrophages but not synovial sublining macrophages display an IL-10 polarized-like phenotype in chronic synovitis. Arthritis Res. Ther..

[68] Barbosa J.N., Pereira Vasconcelos D., Mozafari M. (2020). Chapter 3—Macrophage response to biomaterials. Handbook of Biomaterials Biocompatibility.

[69] Zhang B., Yao G., Zhang Y., Gao J., Yang B., Rao Z., Gao J. (2011). M2-polarized tumor-associated macrophages are associated with poor prognoses resulting from accelerated lymphangiogenesis in lung adenocarcinoma. Clinics.

[70] Bögels M., Braster R., Nijland P.G., Gül N., van de Luijtgaarden W., Fijneman R.J., Meijer G.A., Jimenez C.R., Beelen R.H., van Egmond M. (2012). Carcinoma origin dictates differential skewing of monocyte function. Oncoimmunology.

[71] Patel U., Rajasingh S., Samanta S., Cao T., Dawn B., Rajasingh J. (2017). Macrophage polarization in response to epigenetic modifiers during infection and inflammation. Drug Discov. Today.

[72] Mantovani A., Marchesi F., Malesci A., Laghi L., Allavena P. (2017). Tumour-associated macrophages as treatment targets in oncology. Nat. Rev. Clin. Oncol..

[73] Zhang H., Costabel U., Dai H. (2021). The Role of Diverse Immune Cells in Sarcoidosis. Front. Immunol..

[74] Locke L.W., Crouser E.D., White P., Julian M.W., Caceres E.G., Papp A.C., Le V.T., Sadee W., Schlesinger L.S. (2019). IL-13-regulated Macrophage Polarization during Granuloma Formation in an In Vitro Human Sarcoidosis Model. Am. J. Respir. Cell Mol. Biol..

[75] Zhang X., Liu Y., Jiang M., Mas-Rosario J.A., Fedeli S., Cao-Milan R., Liu L., Winters K.J., Hirschbiegel C.M., Nabawy A. (2024). Polarization of macrophages to an anti-cancer phenotype through in situ uncaging of a TLR 7/8 agonist using bioorthogonal nanozymes. Chem. Sci..

[76] Akhtari M., Quesada J.R., Schwartz M.R., Chiang S.B., Teh B.S. (2014). Sarcoidosis presenting as metastatic lymphadenopathy in breast cancer. Clin. Breast Cancer.

[77] Kobayashi N., Nakamura R., Kurishima K., Sato Y., Satoh H. (2010). Sarcoidosis and lung cancer. Acta Medica.

[78] Sacchi S., Kantarjian H., O’Brien S., Cohen P.R., Pierce S., Talpaz M. (1995). Immune-mediated and unusual complications during interferon alfa therapy in chronic myelogenous leukemia. J. Clin. Oncol..

[79] Su R., Nguyen M.L., Agarwal M.R., Kirby C., Nguyen C.P., Ramstein J., Darnell E.P., Gomez A.D., Ho M., Woodruff P.G. (2013). Interferon-inducible chemokines reflect severity and progression in sarcoidosis. Respir. Res..

[80] Yao M., Funk G.F., Goldstein D.P., DeYoung B.R., Graham M.M. (2005). Benign lesions in cancer patients: Case 1. Sarcoidosis after chemoradiation for head and neck cancer. J. Clin. Oncol..

[81] Esendağlı D., Köksal D., Sarınç Ulaşlı S., Emri S. (2023). Malignancy and sarcoidosis: A single center experience from Turkey. Acta Medica.

[82] Huh J.Y., Moon D.S., Song J.W. (2022). Sarcoid-like reaction in subjects with malignant tumors: Long-term clinical course and outcomes. Front. Med..

[83] Gkiozos I., Kopitopoulou A., Kalkanis A., Vamvakaris I.N., Judson M.A., Syrigos K.N. (2018). Sarcoidosis-Like Reactions Induced by Checkpoint Inhibitors. J. Thorac. Oncol..

[84] Paydas S. (2021). Sarcoid-like reaction in cases treated by checkpoint inhibitors. Med. Oncol..

[85] Brito-Zerón P., Pérez-Alvarez R., Feijoo-Massó C., Gracia-Tello B., González-García A., Gómez-de-la-Torre R., Alguacil A., López-Dupla M., Robles A., Garcia-Morillo S. (2021). Coexistence of immune-mediated diseases in sarcoidosis. Frequency and clinical significance in 1737 subjects. Jt. Bone Spine.

[86] Brito-Zerón P., Kostov B., Superville D., Baughman R.P., Ramos-Casals M. (2019). Autoimmune Big Data Study Group. Geoepidemiological big data approach to sarcoidosis: Geographical and ethnic determinants. Clin. Exp. Rheumatol..

[87] Akaike G., Itani M., Shah H., Ahuja J., Yilmaz Gunes B., Assaker R., Behnia F. (2018). PET/CT in the Diagnosis and Workup of Sarcoidosis: Focus on Atypical Manifestations. Radiographics.

[88] Aoki H., Miyazaki Y., Anzai T., Yokoyama K., Tsuchiya J., Shirai T., Shibata S., Sakakibara R., Mitsumura T., Honda T. (2023). Deep convolutional neural network for differentiating between sarcoidosis and lymphoma based on [^18^F]FDG maximum-intensity projection images. Eur. Radiol..

[89] Shapouri-Moghaddam A., Mohammadian S., Vazini H., Taghadosi M., Esmaeili S.A., Mardani F., Seifi B., Mohammadi A., Afshari J.T., Sahebkar A. (2018). Macrophage plasticity, polarization, and function in health and disease. J. Cell Physiol..

[90] Wang S., Liu R., Yu Q., Dong L., Bi Y., Liu G. (2019). Metabolic reprogramming of macrophages during infections and cancer. Cancer Lett..

