# Supplementary material for: Sarcoidosis and Cancer: The Role of the Granulomatous Reaction as a Double-Edged Sword
Source: J Clin Med. 2024 Sep 4;13(17):5232. doi: 10.3390/jcm13175232 (PMC11396756; doi:10.3390/jcm13175232)
Supplement: Supplementary file 1 [file jcm-13-05232-s001.zip › jcm-3097699-supplementary.pdf]

Supplementary Table 1

Diseases	Tot N.	Smoke (N.) 0	1	2	NA	Fever
Sarcoidosis (S)	251	146	11	74	20	15
S + Breast Cancer	11	9		2		3
S + Melanoma	4	3		1		
S + Hodgkin Lymphoma	4	2*	1	1		
S + Waldenström's macroglobulinemia	1	1				
S + Thyroid Cancer	3	2		1		
S + Endometrial Cancer	3	1	1	1		
S + Ovarian Cancer	2	1		1		
S + Kidney Cancer	1			1		
S + Adenocarcinoma	1			1		
S + Choriocarcinoma	1	1				1
S + Cervical Cancer	1	1				
S + Rectum cancer	1	1				
S + Myeloproliferative neoplasm	1		1			
S + Seminoma	1			1		
S + Urothelial Cancer	1	1				

*ex-passive smoker

Fisher's exact T -test fever in sarcoidosis vs S+Cancer group p = 0.2745 (not significant)
Fisher's exact T -test smoke in sarcoidosis vs S+Cancer group (all subjects 1+2) p= 1 (not significant)

Legend

0	no smoker
1	active smoker
2	ex-smoker
NA	not available
